# The Evaluation and Use of a Food Frequency Questionnaire Among the Population in Trivandrum, South Kerala, India

**DOI:** 10.3390/nu12020383

**Published:** 2020-01-31

**Authors:** Amrita Vijay, Leena Mohan, Moira A. Taylor, Jane I. Grove, Ana M. Valdes, Guruprasad P. Aithal, K.T. Shenoy

**Affiliations:** 1 Nottingham Digestive Diseases Centre, School of Medicine, University of Nottingham, Nottingham NG7 2UH, UK; moira.taylor@nottingham.ac.uk (M.A.T.); Jane.Grove@nottingham.ac.uk (J.I.G.); Guru.Aithal@nottingham.ac.uk (G.P.A.); 2 Department of Twin Research and Genetic Epidemiology, King’s College London, London SE1 7EH, UK; 3 Population Health Research Institute, Trivandrum, Kerala, 695011, India; leenakb@yahoo.com (L.M.);; 4 School of Life Sciences, Faculty of Medicine and Health Sciences, University of Nottingham, Nottingham, NG7 2TQ, UK; 5 National Institute for Health Research (NIHR) Nottingham Biomedical Research Centre, Nottingham University Hospitals NHS Trust and the University of Nottingham, Nottingham NG7 2UH, UK; Ana.Valdes@nottingham.ac.uk; 6 Division of Rheumatology, Orthopedics and Dermatology, School of Medicine, University of Nottingham, Nottingham, NG7 2UH, UK

**Keywords:** food frequency questionnaire, food diary, validation, dietary analysis, India, dietary intake, dietary pattern

## Abstract

Dietary record tools such as food frequency questionnaire (FFQ) and food diaries (FD) are the most commonly used choices for assessing dietary intakes in most large-scale epidemiological studies. The authors developed a self-administered 360-item food frequency questionnaire (FFQ) to assess dietary intakes amongst a population-based cohort in South Kerala. In the validation study (*n* = 460), the data were collected using FFQs that were administered on three different occasions which were then compared to 7-day food records. The intake of foods and nutrients was higher as determined by the FFQ than that assessed using food records. Spearman correlations for macro-nutrients ranged from 0.72 for protein to 0.61 for carbohydrates and for micronutrients, from 0.71 for vitamin B6 to 0.34 for magnesium. The correlation was improved with energy-adjusted nutrient intakes. On average, the exact agreement for the macronutrients ranged from 48.2% to 57.1%, and that for micronutrients ranged from 66.7% to 41.9%, with the median percentage of 49.58%. The authors conclude that the FFQ has an acceptable reproducibility, however, there was a systematic trend towards higher estimates with the FFQ for most nutrients compared to the FD records.

## 1. Introduction

In population-based epidemiologic studies, dietary intake is commonly assessed using dietary assessment methods, such as food diaries (FD), food frequency questionnaires (FFQs) and 24-hour dietary recalls [1]. In particular, FFQs have been widely used in large-scale population-based studies owing to easy administration, less burden on participants and staff, and low cost compared to other assessment methods [2]. FFQs consist of a list of food items with response categories to indicate the usual frequency of consumption over a certain time and estimated total energy and nutrient intakes are calculated by frequency of consumption of each food item, with consideration of portion size. Estimated total energy and nutrient intakes are calculated as the product of the frequency of consumption of each food item, portion size and the energy yield or nutrient composition [3,4]. Food frequency questionnaires have been widely used to assess the nutrient intake across populations for epidemiological purposes and to assess the degree of association with patterns of disease such as chronic and non-communicable diseases including cancer.

Although a number of methods have been used to assess usual dietary intake at the population level [5], the accuracy and reliability of measuring diet still present an on-going challenge [6]. The 24-hour recalls have been widely used, however, depending on the degree of between-day variability in the diet, multiple collections must be made to reflect the habitual diet. This could impose a burden on participants and their economic constraints make them inapplicable for most large epidemiological studies. Food diaries have been considered as the gold standard of dietary methods mainly due to the quality of the dietary data obtained. As food and their quantities are recorded as and when consumed, this addresses memory issues and does not rely on portion size estimations. On the contrary, FFQs are relatively inexpensive, put less burden on the respondents, and do not require trained interviewers [7]. Therefore, they represent the most commonly used tools in epidemiological studies [2]. However, due to lower accuracy, the information collected by FFQs needs to be compared with information collected by a more accurate dietary assessment method. This will be a measure of the relative validity of the FFQ in comparison with a reference method such as the food diary.

India has a diverse dietary pattern with a wide variety of food being consumed and varied recipes, with methods of cooking and portion sizes varying across different regions and communities. Over the past three decades, there have been significant changes in the type of food and patterns of consumption involving both traditional as well as modern food and in parallel, there has been a steady increase in diet-related non-communicable diseases [8,9,10]. Studies on diet and health can be performed economically using dietary tools such as the FFQ and there is a clear need for one that is suitable to evaluate dietary patterns in communities in India. Although various FFQs are widely used in epidemiological studies in the West, very few have been developed and tested to suit the broad and wide diversity of dietary intakes across various regions in India. A reliable and validated FFQ would be able to evaluate food consumption across heterogeneous socio-demographic populations with variable incomes, class and religion [11,12,13], as well as facilitating the exploration of links between dietary intakes and health outcomes.

The current study was the first to validate a FFQ that was designed to assess the dietary intake of individuals from the region of South Kerala against a 7-day FD. The study also aimed to explore differences in habitual dietary intakes that were attributed by body mass index (BMI), age, gender, education and social class.

## 2. Materials and Methods

### 2.1. Population Setting and Participant Recruitment

The study was a cross-sectional one among the rural and urban population in Trivandrum District of South Kerala, India. The study population comprised of individuals from rural and urban sectors of Trivandrum, South Kerala. Households in the rural and urban sectors were selected randomly using the local voter’s list and household visits were scheduled to recruit participants into the study. The household visits included briefing the participants regarding the study by providing them with a study information sheet. Participant consent was either obtained on the same day or at a later date. Any member (>18 years of age) was allowed to take part, however, only one person per household was allowed to enroll in the study. A total of 460 individuals (204 from the urban and 256 from the rural population of the District of Trivandrum) were recruited into the study (ensuring adequate representation of different socioeconomic groups and religions) in order to validate dietary intakes recorded using the FFQ against the 7-day FD.

The socio-economic data were captured using previously published socio-economic status questionnaires [14]. This research study was approved by Sree Gokulam Medical College and Research Foundation Institutional Ethics Committee (Ref:04/36/01/2013).

### 2.2. Dietary Data Collection

Trained nutritionists interviewed the participants at their homes for the FD. Prior to completion of the FFQs, the participants underwent training on portion size estimation wherein they were shown the standard measures of known weight for each food type and were asked to use similar household measures to help them estimate their portion size as a multiple of the standard measure. The FFQ was administered on three separate days (i.e., days 1, 5 and 9) whereas the FD was completed over a period of seven consecutive days (i.e., days 2, 3, 4, 5, 6, 7 and 8). Participants reported the portion sizes and frequency of consumption of food items based on daily, weekly and monthly intakes over the past year. The reported frequency of intake for each food item on the FFQ was multiplied by the reported portion size and its respective energy yield and nutrient composition which was calculated and derived from the Nutritive value of Indian Foods Database [15] which served as a reference database for both the FFQ and Food diary data. The foods reported by the participants that were not in the Nutritive value of Indian Food Database were referred to the Indian Food Composition Database for resolution [16].

### 2.3. Nutrient Intake Assessment using the FFQ

The FFQ consisted of 360 food items that recorded intakes based on the following categories: daily, weekly, monthly, occasional/seasonal and never. Food items were determined based on a previously published FFQ consisting of 81 food items and validated in a rural district of south Kerala [12]. The remainder of the food items was determined via interviews conducted on 150 individuals from specific urban and rural districts in Trivandrum. Interviews were conducted by household visits performed by trained nutritionists. The 360 food items were included as these encompassed all types of foods consumed in the region of Trivandrum, South Kerala, at the time of the study. The FFQ consisted of 315 composite food items (i.e., food items containing one or more ingredients) and 45 simple food items. The nutritive value of each ingredient was derived from the Nutritive Value of Indian Foods Database [15], which provides a comprehensive breakdown of nutritional information for 591 food items which are expressed per 100 grams. The macro and micronutrient values for each ingredient used in the recipe was derived from the reference database and calculated in proportion to the amount used in the recipe. By adding the nutritive value of each ingredient, the nutritive value of the recipe was then calculated.

### 2.4. Statistical Analysis

Associations between nutrient intakes from the first administration of the FFQ and the mean of the 7-day FD were compared with Spearman rank correlations, one-way ANOVA to test for significance followed by paired t-tests, cross-classification and Bland–Altman plots. Bonferroni correction was applied where multiple testing was performed. Individuals who fell outside of the energy intake range (men <3347 kJ or >17573 kJ, women <2510 kJ or >14644 kJ, 1 cal = 4.184 kJ) were excluded as part of the analysis. Simple linear regression was used to assess the linear agreement between the FFQ-derived nutrient scores and arithmetic average of those obtained from the 7-day FD records. The agreement between the two methods was evaluated using the Bland–Altman method [17]. Cross-classification analysis was used to assess the percentage of agreement and the ability of the FFQ to classify participants into similar quintiles of nutrient intake based on the results from the 7-day food records. The data from the first administration of the FFQ was used for the calculation of nutrient intake estimates and validation statistics. In order to reduce the effect of overestimating or underestimating food intake, energy adjustment was used using the residual method.

To identify factors associated with the validity of FFQ intake estimates, multivariable regression analysis was performed with the difference in nutrient intakes between dietary methods as the dependent variable and personal characteristics of participants as independent variables. The regression coefficient (R^2^) was calculated to quantify the extent to which the independent variables accounted for total variation in the difference in intakes. All statistical analyses were carried out in Prism (version 8.0, San Diego, USA) and R v3.5.2 (Vienna, Austria).

## 3. Results

### 3.1. Population Demographics

The demographics are shown in Table 1. Participants were predominantly female (66%), aged 31–40 (30%) and the majority were educated to a high school degree or higher (85%).

### 3.2. Mean Nutrient Intakes Across Income, Religion and Domicile

**Figure 1:**
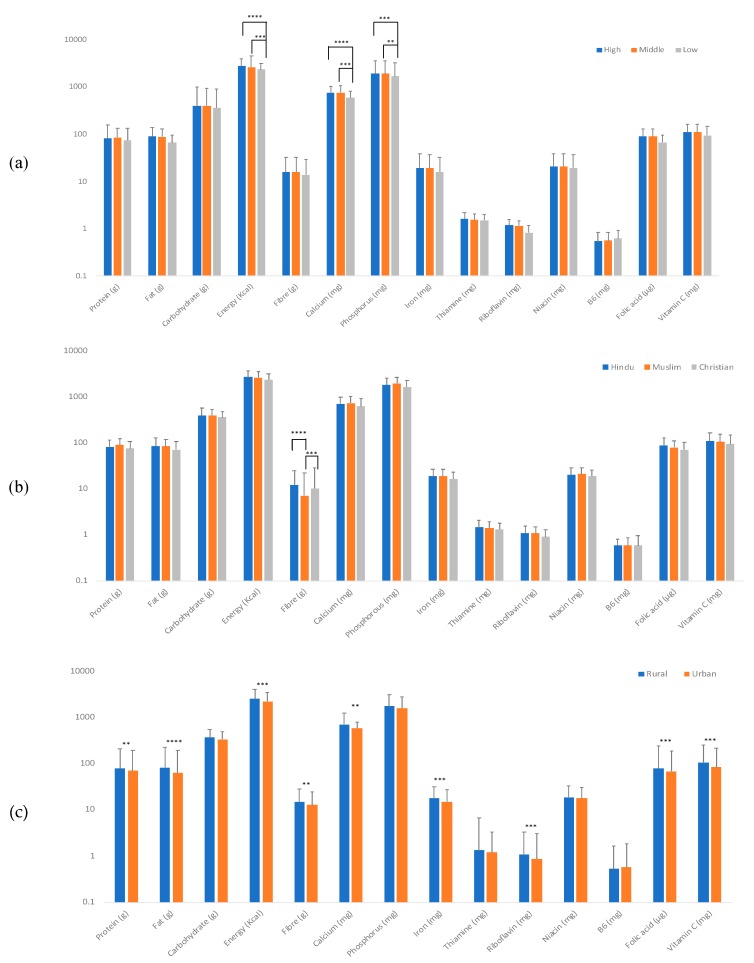
Mean nutrient intakes derived from the first FFQ across (**a**) income, (**b**) religion, (**c**) domicile. * Bonferroni adjusted *p* values. ** *p* < 0.005; *** *p* < 0.0005; **** *p* < 0.0001.

The mean nutrient intake across socio-economic groups was estimated from the first administered FFQ. Overall, in the mean intake of energy, minerals such as calcium and phosphorous were significantly lower in the low-income group compared to the middle and high-income groups (Figure 1a). The mean nutrient intake across different religions showed no significant differences for all nutrients with the exception of fibre which was significantly lower in Muslims compared to Hindus and Christians (Figure 1b). However, significant differences were seen in the intake of most nutrients between domiciles (i.e., between urban and rural) as shown in Figure 1c.

### 3.3. Associations in Nutrient Intakes between the FFQ and 7-Day Food Diary

There were significant correlations between most nutrients from both methods (range 0.346–0.729). The correlations for energy-adjusted nutrients intake ranged from 0.413 to 0.810. The lowest non-significant correlations (≤0.40) were for magnesium, manganese, carotene, which remained non-significant even with energy-adjusted intakes. However, vitamin C was found significant with energy adjustment. The highest significant correlations after energy adjustment were for protein, fibre and minerals such as phosphorous and sodium (Table 2).

### 3.4. Associations between Personal Characteristics and Difference in Reported Intakes between the FFQ and 7-Day Food Diary

Table 3 shows the associations between personal characteristics of individuals and the difference in estimated intake between the first administration of the FFQ and 7-day food diary. *R*^2^ ranged from as low as 4% up to 32%. None of the personal characteristics were significant in the models for those with the lowest *R*^2^ for nutrients such as sulfur, carotene, sodium, potassium, copper, manganese and molybdenum. Sex was significant for eight of the nutrients with the difference in reported intakes being larger among women than men for protein, fibre and calcium, whereas the difference was smaller for the intake of minerals and vitamins. Age was significant for intakes of total energy and calcium, and socio-economic status was significant for intakes of fibre, iron, niacin and sodium. BMI was found not significantly associated with reported intake for any nutrient, however, showed negative associations with the reported intakes of carbohydrates, fat and some minerals.

### 3.5. Bland–Altman Analysis

**Figure 2:**
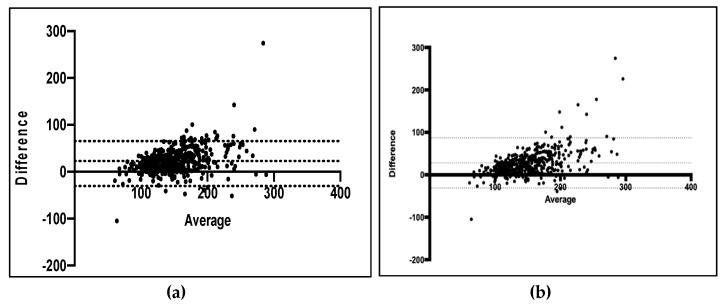
Differences between nutrient intake estimated from the food-frequency questionnaire (first administration) and from the 7-day food records plotted against the mean from the two methods (*n* = 460). For each participant, the difference in energy adjusted intakes between the FFQ (first administration) and the average of the 7-day food records is plotted against the mean intake from the two methods for: (**a**) Macronutrients, mean difference = 23.78 (95% CI of −30.27, 68.96), (**b**) energy (kcal), mean difference = 393.2 (95% CI of −441.3, 1228) with FFQ overestimating the nutrient intake compared to food diary.

When considering if the methods agreed for individuals, the differences in nutrient intake between the FFQ and the 7-day food records were plotted against the mean nutrient intakes of the two methods (Table 4). Figure 2 shows the plots for macronutrients and energy intakes. Positive differences in the average discrepancy between the two methods indicate an overestimation of nutrient intake by the FFQ. The larger the value of the bias (wider the limits of agreement as indicated by the 95% CI), the larger the extent of overestimation by the FFQ. For macronutrients such as carbohydrates and fats and a range of micronutrients, there was some bias towards a positive difference, suggesting that the FFQ provides a higher intake of certain macro and micronutrients as compared with the food diary. Similar results were obtained for most of the nutrients, as summarized in Table 4. Overall, the Bland–Altman plots showed that there was a systematic trend towards higher estimates with the FFQ for certain nutrients compared with the food diary records. The results of the analysis are tabulated below (Table 4).

Figure 2. displays the findings of the Bland–Altman analysis for macronutrients and total energy intakes. In these Bland–Altman plots, mean intake from both the dietary method was plotted in X-axis, and the difference in intakes of the participants was plotted in Y-axis.

### 3.6. Cross Classification Analysis

Agreement within quartiles between the 7-day food records and FFQ is shown in Table 5. Subjects were classified into the same or adjacent quartiles or misclassified into extreme or intermediate quartiles. On average, more than 70% of the subjects were classified into the same or adjacent quartiles with less than 10% misclassified into extreme or intermediate classes. The agreement for the macronutrients ranged from 41.2% to 57.1% for crude and from 50.4% to 59.3% for energy-adjusted. On the contrary, miss-classifications in opposite extreme quartiles were found to be high for carbohydrates, vitamin C and carotene even after energy adjustment. A similar trend was observed for the misclassification in the intermediate quartiles.

## 4. Discussion

We have developed an FFQ to include a wide range of food items that are usually consumed and is representative of the common dietary patterns of the population in Kerala, India. To our knowledge, this 360-item FFQ is the largest to be validated for use in this region [12,18]. The validated FFQ could be used as a suitable tool to identify important dietary intake patterns in the region of Kerala as part of future studies. This serves as an important basis for designing epidemiological studies in this specific region, where there is a growing concern regarding metabolic diseases such as Type 2 diabetes, cardiovascular disease and non-alcoholic fatty liver disease (NAFLD). In the present study, 460 participants completed all of the questionnaires (i.e., FFQ and 7-day food diary). The minimum sample size for the validation of dietary questionnaires is suggested at 100–200 participants [4,19]. Our study represented an appropriate sample size to assess the reliability of the FFQ.

In the population of Kerala, South India, there is a possibility of variation in nutrient intake in different social class and religion with seasonal variation been studied previously by Hebert et al. [11]. In comparison with the 7-day food diary, the FFQ overestimated unadjusted nutrients, as seen in previous studies [12].

The average intake of nutrients was also associated with socio-economic factors such as income. In the current study, we found that there were higher intakes of both macro and micronutrients amongst the high- and middle-income sectors compared with the lower-income sector. In particular, significant differences were seen in total energy intake in high- and middle-income sectors compared with the lower-income group. Considering the potential cumulative impact of these differences in energy intake in the long-term, the importance of these associations should be investigated in the future. In general, most of the studies in India analyze prices and expenditure as one of the important factors affecting food consumption patterns and less attention has been paid to socio-economic and regional variables, which may incur differences in food consumption patterns. On average, people belonging to higher income class are associated with healthier dietary patterns, which includes fruits, vegetables, oil and meat consumption. The higher income class people consume more of these food items than their lower-class counterparts probably because with higher income, socio-economic status increases which results in more knowledge and awareness of health and healthy food items [20,21,22]. In addition, it has been found that unemployment or low income becomes a barrier in the purchase of fruit and vegetable consumption as reported previously [23,24]. The only intake that showed any differences in intake between the religions was fibre. This could be due to variations in food choices and habitual dietary patterns of specific religious classes [25,26].

The average of the 7-day FD was considered to correlate with the FFQ derived nutrient values for validation. The correlation coefficient we observed between the FFQ and 7-day FD (0.34 to 0·72) were similar to those reported previously in a validation study conducted in Kerala (ranging from 0.32 to 0.61) [12] and in Gujarat (ranging from 0.55 to 1.00) [13]. Additionally, some other studies, done with population groups in a similar region of India, also demonstrated a range of coefficients, which appeared to be similar to our range [18,27,28]. In the present study, the highest correlation was observed for macronutrients such as proteins, whereas lower correlations were observed for trace elements such as magnesium, manganese and vitamins such as vitamin C and carotene, which have been reported previously [29,30]. Since these nutrients are not concentrated in a majority of foods, they may tend to have high within-person variability and lower correlation co-efficient in validation studies, as also reported previously [13,30].

Adjustment of energy improved the agreement of nutritional intakes that were estimated by the FFQ compared to the 7-day FD. After adjusting for energy, there was an improvement in the overall range of correlation coefficients. However, certain macronutrients such as carbohydrates and fats and micronutrients such as calcium, phosphorous, magnesium, choline, carotene, potassium were overestimated by the FFQ compared to the food diary. Copper and zinc were underestimated by the FFQ compared to the food record. According to the multiple regression model, 25% of the variation in the difference between the two assessment methods is explained by sociodemographic independent variables. Sex was a significant explanatory variable for most of these with women over-reporting intakes compared to men as observed in previous studies [31]. The influence of income on dietary fibre intake has been reported previously where lower family income was associated with lower dietary fibre intakes amongst adults [32].

There are some substantial strengths to our study. Firstly, the validity of the FFQ was evaluated with a comprehensive range of tests, including correlations coefficients and cross-classification in conjunction with the Bland–Altman method. The Bland–Altman method has been preferred over correlation analysis as a method to evaluate the reproducibility and validity of an FFQ. Furthermore, the sample size of the present study was large enough to allow for the estimation of the limits of agreement from the Bland–Altman analysis as a component of the evaluation of the validity of the FFQ. In addition, participants received guidance regarding portion size before the FFQs were administered to assist self-administration. This, we think, is the strength of the study avoiding underreporting and improving consistency. The guidance on portion size will be implemented as a pre-requisite for all future administrations of the current validated FFQ.

We note that there are some limitations to our study. The parallel administration of the FFQ and FD could have influenced the memory and reporting patterns of the participants and thereby resulted in some degree of misreporting or overestimation of nutrient intakes. The length of the FFQ could have resulted in participant fatigue as opposed to shorter FFQs that have been used previously. This may also have contributed to the over-estimation of nutrient intakes we describe. The sources of error in the FFQ could be due to restrictions imposed by a fixed list of foods, seasonal variations, memory, perception of portion sizes and interpretation of questions [33,34]. In addition, the authors would also like to acknowledge the limitation of using the food diary as the reference method. Although FD and records are commonly used as the standard reference tool for most validation studies, the prevalence of under or over-reporting is a common issue. Most consistent differences in under and over-reporting are found between men and women, and between groups differing in BMI where obese individuals normally under-report their dietary intakes [35]. Since the food diary captures information over a short period of time (i.e., seven days), the dietary intakes recorded would reflect to some extent the foods that are commonly available during that particular season [11]. In addition to the above, the current study also lacked biomarkers for the cross-validation of nutrient intakes which could be considered in future work.

In conclusion, the development and validity of the current FFQ is an important first step that allows us to implement this as a tool in epidemiological studies to assess food intakes, eating behaviors and correlations to disease phenotypes amongst the population of South Kerala, India. Potential for application of this FFQ across the state of Kerala should be assessed in the future.

## Figures and Tables

**Table 1:** Summary of the socio-demographic characteristics of study participants (*n* = 460).

Demography	Frequency	%
**Gender**		
Male	157	34
Female	303	66
Age (years)		
<30	66	14
31–40	140	30
41–50	108	23
51–60	81	18
>60	65	14
**BMI**		
Underweight (<18.5 kg/m^2^)	33	7.2
Normal (18.5–22.9 kg/m^2^)	240	52.2
Overweight (23.0–24.9 kg/m^2^)	136	29.6
Obese (≥25 kg/m^2^)	51	11.1
**Monthly Income (Rupees)**		
<1000	105	23
1001–3000	131	28
3001–6000	112	24
>6000	112	24
**Education Status**		
Primary or below	69	15
High school	233	51
Higher secondary	69	15
Graduate or above	89	19
**Domicile**		
Rural	256	55
Urban	204	45
**Religion**		
Hindu	297	65
Muslim	77	17
Christian	86	18

**Table 2:** Correlation of nutrients estimated by the first food frequency questionnaire (FFQ) and 7-day food diaries (FD).

Nutrient	FD		FFQ		Difference (FFQ − FD) ± SD	FFQ:FD (%)	Spearman Coefficient (Unadjusted (r))	Spearman Coefficient (Energy Adjusted (R))
	Mean	SD	Mean	SD				
Protein (g)	62.154	18.59	81.68	34.59	19.52 ± 25.57 ***	1.41	0.729 ****	0.810 ***
Minerals (g)	9.878	2.86	13	4.94	3.12 ± 3.574 ***	1.30	0.685 ****	0.617 ****
Fibre (g)	11.439	3.8277	15.32	6.77	3.88 ± 2.782 ***	1.32	0.659 ****	0.719 ****
Carbohydrate (g)	294.308	75.309	391.6	153.89	97.29 ± 125.3 ***	1.53	0.610 ****	0.518 ****
Energy (kcal)	1980.38	510.561	2635.24	1001.03	654.86 ± 771.1 ***	1.47	0.653 ****	n.a.
Calcium (mg)	555.908	198.589	704.18	297.99	148.27 ± 2124 ***	1.18	0.700 ****	0.673 ****
Phosphorus (mg)	1382.07	378.560	1841.33	696.25	459.26 ± 775.9 ***	1.38	0.704 ****	0.712 ****
Fat (g)	61.6898	23.011	82.48	39.98	20.79 ± 29.59 ***	1.29	0.681 ****	0.510 ***
Riboflavin (mg)	0.8389	0.3033	1.06	0.44	0.22 ± 1.691	1.14	0.719 ****	0.656 ***
Chloride (mg)	123.474	58.758	163.11	89.23	39.63 ± 75.93 ***	1.15	0.537 ****	0.598 ****
Sulfur (mg)	110.734	70.087	143.79	96.35	33.05 ± 72.15 ***	1.05	0.664 ****	0.619 ****
Magnesium (mg)	375.306	199.683	481.19	181.64	105.88 ± 218.2 ***	0.70	0.346	0.413
Choline (mg)	167.922	77.856	212.45	97.18	44.52 ± 87.26 ***	0.98	0.519 ****	0.578 ****
Vitamin C (mg)	81.758	45.350	105.84	54.58	24.08 ± 50.8 ***	0.92	0.495	0.612 **
Thiamine (mg)	1.03178	0.3113	1.4	0.55	0.368 ± 1.453 ***	1.30	0.673 ****	0.553 ***
Iron (mg)	13.4568	4.1980	18.28	7.82	4.82 ± 6.241 ***	1.37	0.643 ****	0.612 ****
Niacin (mg)	14.728	4.0433	20.39	7.85	5.66 ± 6.285 ***	1.19	0.613 ****	0.654 ****
Carotene (μg)	1173.29	2034.17	1201.26	876.2	27.97 ± 1877	1.40	0.385	0.418
B6 (mg)	0.4334	0.169	0.58	0.28	0.14 ± 1.809	0.42	0.712 ****	0.678 ***
Folic Acid (μg)	62.385	26.554	82.67	38.02	20.28 ± 24.65 ***	1.23	0.716 ****	0.617 ***
Sodium (mg)	156.898	76.954	196.55	118.98	39.652 ± 88.76 ***	1.08	0.666 ****	0.719 ****
Potassium (mg)	754.281	346.42	970.77	495.89	216.48 ± 353.1 ***	1.23	0.703 ****	0.652 ***
Copper(mg)	1.512	6.775	1.65	1.03	0.138 ± 21.88	1.11	0.641 ****	0.562 ***
Manganese (mg)	3.252	1.099	4.45	1.85	1.198 ± 2.084	0.14	0.354	0.487
Molybdenum (mg)	0.336	0.115	0.44	0.18	0.104 ± 1.572	1.23	0.602 ****	0.586 ****
Zinc (mg)	5.5434	1.673	7.48	2.88	1.9366 ± 2.542	1.19	0.513 ****	0.617 ****
Chromium (mg)	0.0389	0.014	0.05	0.02	0.0111 ± 1.701	1.27	0.673 ****	0.598 ****

*** *p* < 0.001; **** *p* < 0.0001. SD, Standard deviation; n.a., not applicable; (r) unadjusted for energy intakes, (R) adjusted for energy intake

**Table 3:** Association between factors and differences in intake estimates (regression coefficients) using multiple regressions with nutrients as the dependent variable.

Nutrient	Age ^1^	Sex ^2^	BMI ^1^	Education ^3^	Income ^4^	Model R^2^ Values
Protein (g)	0.001	0.535 ^++^	0.003	0.038	0.288 ^+^	0.25
Minerals (g)	0.004	0.142	−0.028	0.097	0.230	0.17
Fibre (g)	0.009	0.786 ^+^	0.003	0.086	0.412 ^+^	0.15
Carbohydrate (g)	0.004	0.484 ^+^	−0.015	0.107	0.062	0.25
Energy (kcal)	0.009 ^+^	−0.230	0.038	0.093	0.400	0.12
Calcium (mg)	0.008 ^+^	0.529 ^+^	−0.009	0.049	−0.361	0.26
Phosphorus (mg)	−0.003	0.452 ^+^	0.033	−0.118	−0.271	0.15
Fat (g)	0.006	0.438	−0.009	0.059	0.541	0.12
Riboflavin (mg)	0.021	0.274	0.033	0.057	0.312	0.19
Chloride (mg)	−0.005	0.452 ^++^	0.004	0.010	0.242	0.20
Sulfur (mg)	−0.005	0.270 ^+^	0.018	−0.139	−0.571	0.07
Magnesium (mg)	0.027	0.152	0.062	−0.023	−0.136	0.28
Choline (mg)	0.010	0.290	0.034	−0.107	0.002	0.14
Vitamin C (mg)	−0.006	0.312	0.026	−0.023	−.153	0.10
Thiamine (mg)	0.011	0.443	0.011	−0.064	0.026	0.31
Iron (mg)	−0.006	0.152 ^++^	0.034	0.106	0.581 ^+^	0.32
Niacin (mg)	−0.014	0.290	0.018	0.009	0.665 ^+^	0.14
Carotene (μg)	0.004	0.380	0.054	−0.126	−0.373	0.07
B6 (mg)	0.011	0.098	0.034	−0.235	0.221	0.23
Folic Acid (μg)	0.007	0.152	0.040	0.129	0.067	0.10
Sodium (mg)	−0.007	0.089	0.020	−0.168	0.722 ^+^	0.07
Potassium (mg)	−0.004	−0.059	0.011	0.299 ^+^	0.306	0.08
Copper (mg)	0.014	−0.295	−0.012	0.163	−0.043	0.08
Manganese (mg)	0.005	−0.324	−0.002	0.255 ^+^	−0.077	0.06
Molybdenum (mg)	0.002	−0.092	0.071	−0.003	0.583	0.04

Multiple regression model with nutrients as the dependent variables. Factors associated with difference in (energy adjusted) daily grams of nutrient intakes from the FFQ (first administration) and 7-day food diary: Regression models were multivariable, with each factor adjusted for all others (^+^ *p* < 0.05; ^++^ *p* < 0.001). ^1^ Continuous variable. ^2^ Reference category: Men. ^3^ Reference category: Primary or below. ^4^ Reference category: <1000 Rupees/month.

**Table 4:** Bland–Altman analysis of nutrients between the FFQ and 7-day food diary.

Nutrient	Bias *	SD	95% CI
Protein (g)	9.45	10.21	−12.67, 26.55
Minerals (g)	1.53	2.17	−2.03, 6.11
Fibre (g)	5.21	3.22	−2.53, 5.34
Carbohydrate (g)	35.15	12.3	−34.22, 172
Energy (kcal)	n.a.	n.a.	n.a.
Calcium (mg)	34.14	22.5	−56.3, 102.4
Phosphorous (mg)	81.4	34.6	−78.4, 140
Fat (g)	18.93	10.45	−37.21, 91.39
Riboflavin (mg)	1.55	0.463	−0.15, 1.32
Chloride (mg)	13.45	21.43	−45.22, 73.7
Sulfur (mg)	11.32	31.45	−52.14, 78.2
Magnesium (mg)	71.42	96.43	−123.4, 221
Choline (mg)	82.32	65.4	−115.1,198.3
Vitamin C (mg)	18.23	21.43	−78.14, 119.56
Thiamine (mg)	0.13	0.14	−0.221, 0.62
Iron (mg)	4.14	5.33	−1.35, 8.44
Niacin (mg)	2.33	4.13	−3.332, 8.33
Carotene (μg)	43.15	11.33	−341, 1555
B6 (mg)	1.14	1.16	−1.228, 2.11
Folic acid (μg)	10.98	11.84	−31.14, 51.66
Sodium (mg)	20.82	31.32	−83.33, 145.8
Potassium (mg)	180.6	146.9	−255.6, 612.5
Copper (mg)	−1.44	21.54	−214.31, 114.14
Manganese (mg)	0.94	1.22	−0.14, 2.11
Molybdenum (mg)	0.16	0.56	−0.65, 0.53
Zinc (mg)	−2.33	1.53	−11.66, 4.78
Chromium (mg)	0.093	0.12	−0.16, 1.32

Analysis performed on energy-adjusted nutrient intake; SD, standard deviation; CI, Confidence interval; n.a., not applicable. * Positive differences in the average discrepancy between the two methods indicate an overestimation of nutrient intake by the FFQ.

**Table 5:** Cross classification of quartiles by 124 food items listed food frequency questionnaire (FFQ) and the 7-day food records.

Nutrient ^*^	FFQ / 7-Day FD							
	Crude Nutrients				Energy Adjusted Nutrients			
	% in the same Quartile	% in Adjacent Quartiles	% in Extreme Quartiles	% in Intermediate Quartiles *	% in the same Quartile	% in Adjacent Quartiles	% in Extreme Quartiles	% in Intermediate Quartiles
Protein (g)	51.3	46.2	2.5	3.6	52.5	43.3	1.8	2.7
Minerals (g)	57.1	41.5	1.4	2.3	59.3	42.6	1.6	1.6
Fibre (g)	52.2	46.8	1.0	2.7	55.3	45.3	0.0	1.8
Carbohydrate (g)	41.2	52.2	6.6	4.6	44.5	51.2	3.4	3.9
Energy (kcal)	42.4	52	5.6	6.2	n.a	n.a.	n.a.	n.a.
Calcium (mg)	61.4	37.4	1.2	3.2	57.4	24.8	2.7	2.8
Phosphorous (mg)	58.4	40.8	0.8	2.8	60.1	38.3	1.7	2.1
Fat (g)	48.9	47.4	3.7	4.1	50.4	48.3	2.1	3.2
Riboflavin (mg)	60.12	37.8	2.0	3.8	58.5	38.2	1.8	2.9
Chloride (mg)	50.1	48.9	1.0	3.1	54.3	43.5	1.8	2.5
Sulfur (mg)	51.9	45.6	2.5	4.2	49.6	50.2	1.7	3.2
Magnesium (mg)	57.2	41.4	1.4	3.8	58.4	40.5	1.2	2.8
Choline (mg)	48.3	50.3	1.4	2.9	49.4	47.3	1.3	2.1
Vitamin C (mg)	57.6	39.7	2.7	2.8	56.4	34.2	2.9	1.8
Thiamine (mg)	45.3	52.6	2.1	3.1	48.3	51.2	1.2	2.2
Iron (mg)	67.6	31.3	1.1	2.6	59.4	38.2	1.0	1.5
Niacin (mg)	51.8	47.2	1.0	2.8	49.6	48.2	1.2	1.8
Carotene (μg)	46.2	49.6	4.2	4.2	45.4	50.4	3.4	3.5
B6 (mg)	60.3	38.5	1.2	2.8	58.3	41.8	1.1	1.8
Folic acid (μg)	62.3	36.2	1.5	3.5	60.4	37.4	1.7	3.1
Sodium (mg)	52.7	46.2	1.1	2.8	53.6	45.3	1.0	1.7
Potassium (mg)	51.4	45.6	3.0	2.9	54.3	42.5	2.7	2.3
Copper (mg)	41.9	56.3	1.8	2.6	42.5	55.3	2.0	2.1
Manganese (mg)	66.7	31.5	1.8	2.9	65.43	32.8	2.0	1.9
Molybdenum (mg)	43.8	56.1	0.1	3.1	44.65	55.3	1.0	2.7
Zinc (mg)	61.2	37.2	1.6	2.8	60.3	35.5	1.3	2.1
Chromium (mg)	67.3	31.8	0.9	6.2	68.4	30.4	0.0	5.4

* Quartiles Q1 versus Q3; n.a., not applicable.
